# Effects of Unexpected Chords and of Performer's Expression on Brain Responses and Electrodermal Activity

**DOI:** 10.1371/journal.pone.0002631

**Published:** 2008-07-09

**Authors:** Stefan Koelsch, Simone Kilches, Nikolaus Steinbeis, Stefanie Schelinski

**Affiliations:** 1 Department of Psychology, University of Sussex, Brighton, United Kingdom; 2 Junior Research Group *Neurocognition of Music*, Max Planck Institute for Human Cognitive and Brain Science, Leipzig, Germany; University of Minnesota, United States of America

## Abstract

**Background:**

There is lack of neuroscientific studies investigating music processing with naturalistic stimuli, and brain responses to real music are, thus, largely unknown.

**Methodology/Principal Findings:**

This study investigates event-related brain potentials (ERPs), skin conductance responses (SCRs) and heart rate (HR) elicited by unexpected chords of piano sonatas as they were originally arranged by composers, and as they were played by professional pianists. From the musical excerpts played by the pianists (with emotional expression), we also created versions without variations in tempo and loudness (without musical expression) to investigate effects of musical expression on ERPs and SCRs. Compared to expected chords, unexpected chords elicited an early right anterior negativity (ERAN, reflecting music-syntactic processing) and an N5 (reflecting processing of meaning information) in the ERPs, as well as clear changes in the SCRs (reflecting that unexpected chords also elicited emotional responses). The ERAN was not influenced by emotional expression, whereas N5 potentials elicited by chords in general (regardless of their chord function) differed between the expressive and the non-expressive condition.

**Conclusions/Significance:**

These results show that the neural mechanisms of music-syntactic processing operate independently of the emotional qualities of a stimulus, justifying the use of stimuli without emotional expression to investigate the cognitive processing of musical structure. Moreover, the data indicate that musical expression affects the neural mechanisms underlying the processing of musical meaning. Our data are the first to reveal influences of musical performance on ERPs and SCRs, and to show physiological responses to unexpected chords in naturalistic music.

## Introduction

During the last two decades, numerous studies have investigated neural correlates of music processing, of which surprisingly few actually used authentic musical stimuli (for exceptions, see, e.g., [1]–[3]). For example, the majority of experiments investigating music-syntactic processing used chord sequences played under computerized control without musical expression, and composed in a fashion which is often hardly reminiscent of natural music (with the purpose to control for acoustical factors, or to present as many stimuli as possible in a relatively short time, e.g. [4]–[14]). With regards to neuroscientific experiments on music-syntactic processing, Koelsch & Mulder [15] used chord sequences recorded from classical CDs, but the irregular chords used to investigate harmonic expectancy violations were produced by a pitch-shift of chords, thus not representing natural music-syntactic violations. Similarly, Besson et al. [16] used sung opera-melodies, but the irregular notes were introduced by the investigators and not originally composed that way (see also Besson & Faïta [17] in that study melodies were played by a computer, and irregular notes were introduced by the investigators). To our knowledge, the only study that has investigated brain responses to irregular chords as composed by a composer, employed chorales from J.S. Bach [18]. However, although that study used the unexpected harmonies originally composed by Bach to investigate music-syntactic processing, the chorales were played under computerized control (without musical expression), thus not sounding like natural music (see also Patel et al. [19], for a study with self-composed music in popular style). It is therefore an open question whether the hypotheses derived from previous neurophysiological studies on music-syntactic processing also apply to natural music.

**Figure 1:**
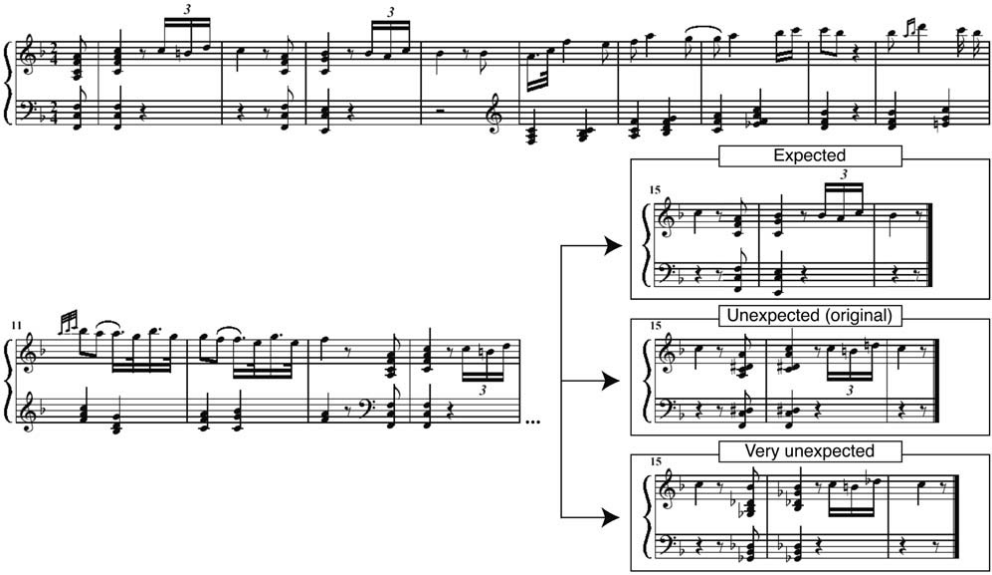
Examples of experimental stimuli. First, the original version of a piano sonata was played by a pianist. This original version contained an unexpected chord as arranged by the composer (see middle panel in the lower right). After the recording, the MIDI file with the unexpected (original) chord was modified offline using MIDI software so that the unexpected chord became expected, or very unexpected chord (see top and bottom panels). From each of these three versions, another version without musical expression was created by eliminating variations in tempo and key-stroke velocities (excerpts were modified offline using MIDI software). Thus, there were six versions of each piano sonata: Versions with expected, unexpected, and very unexpected chords, and each of these versions played with and without musical expression.

In the present study, we used excerpts from classical piano sonatas, played by professional pianists, to investigate music-syntactic processing. The excerpts contained a music-syntactically irregular chord as originally composed by the composer. This allowed to test whether brain responses observed in previous studies in response to music-syntactic irregularities (particularly the early right anterior negativity [ERAN] and the N5 [5], [20]–[23]) can also be observed when listening to an authentic, and expressively played, musical stimulus. For purposes of comparison, additional conditions were created in which the originally unexpected chords (as composed by the composers) were rendered harmonically expected, and harmonically very unexpected (see Figure 1).

Moreover, we also produced non-expressive counterparts of the expressively played musical stimuli. These non-expressive stimuli did not contain any variation in tempo (and all notes were played with the same key-stroke velocity), enabling us to compare ERP responses to unexpected harmonies between conditions in which the music was played expressively, or presented without any expression. So far, no ERP study has investigated the influence of musical performance on music perception, and it is not known if neural correlates underlying the processing of syntactic information are influenced by emotional expression. Previous studies have suggested that both ERAN and N5 reflect cognitive, not affective processes (the ERAN the processing of music-syntactic information, and the N5 processes of harmonic integration; e.g. [24]). Thus it was expected that neither ERAN nor N5 would be influenced by aspects giving rise to emotion, and that they would thus not differ between the non-expressive and the expressive condition.

Nevertheless, it is widely assumed that irregular musical events (such as music-syntactically irregular chords) give rise to emotional responses. Harmonically unexpected chords may lead to surprise or a feeling of suspense [25], [18]. In his classic text on musical meaning and emotion, Leonard Meyer [25] theorized that listeners often have (implicit) expectations of what will happen in the music and, depending on whether these expectations are fulfilled or not, experience relaxation or tension and suspense. A previous study from Steinbeis et al. [18] provided a direct test of this theory, investigating the role of music-specific expectations in the generation of emotional responses in the listener. In that study, unexpected chords elicited not only ERAN and N5 potentials in the EEG, but also an increased skin conductance response (SCR). Because the study from Steinbeis et al. [18] is, to our knowledge, the only study empirically testing a theory about how music evokes emotions (see also [26]), we aimed to replicate the findings of that study. We therefore also recorded SCRs elicited by expected and unexpected chords with the hypothesis that music-syntactically irregular chords (which are perceived as less expected by listeners) elicit a stronger SCR compared to regular chords. In addition to the SCRs, we also recorded the heart rate (HR) to examine whether sympathetic effects elicited by unexpected harmonies can also be reflected in HR changes.

Additionally, our experimental design also allowed us to compare SCRs (and HR) between the expressive and the non-expressive condition. Previous studies have shown that expressive intentions by performers (such as tension and relaxation) are encoded by expressive cues (for example, tempo and loudness) to communicate emotion in a musical performance (for a review, see [27]). Because harmonically unexpected chords are widely seen as a means to produce tension (see above), it was expected that such chords are played by performers in a way that produces an emotional response which is larger than when played without musical expression. Thus, we hypothesized that the SCRs elicited by unexpected (as compared to the SCRs elicited by expected chords) would be larger in the expressive than in the non-expressive condition.

In summary, we investigated ERPs, SCRs, and HR in response to unexpected chords (as composed by classical composers) in a condition in which musical excerpts were played with musical expression by a pianist, and in a condition in which these excerpts were played without musical expression by a computer (without variations in tempo or loudness). We hypothesized that unexpected harmonies would elicit an ERAN and an N5, and that both ERPs would not be influenced by musical expression. Moreover, we hypothesized that unexpected chords would elicit stronger SCRs, and increased HR, compared to expected ones. With regards to musical expression, we hypothesized that SCRs elicited by the expressive chords would elicit stronger SCRs than the non-expressive chords, and that the SCR effect of unexpected chords (i.e., SCRs to expected chords subtracted from SCRs to unexpected chords) would be larger in the expressive than in the non-expressive condition.

## Methods

### Participants

20 individuals (aged 19–29 years, mean 24,7; 10 females) participated in the experiment. Subjects were non-musicians who had not received any formal musical training besides normal school education. All participants had a laterality quotient >90 according to the Edinburgh Handedness Inventory [28]. Written informed consent was obtained, the study was approved by the local ethics committee of the University of Leipzig, and conducted in accordance with the Declaration of Helsinki.

### Stimuli

Stimuli were excerpts of 8 to 16 s duration, taken from 25 piano sonatas composed by L. v. Beethoven, J. Haydn, W.A. Mozart and F. Schubert. Excerpts were chosen such that they contained a harmonically (slightly) irregular, thus unexpected, chord at the end of the excerpt (usually the onset of a change of key, see Figure 1 for an example). Each excerpt was taken from a recording of a longer passage of the respective piano sonata. Passages were played by 4 professional pianists (2 of them female), and recorded using MIDI (musical instrument digital interface) and Cubase SX (Steinberg Media Technologies GmbH, Hamburg, Germany) software.

From the MIDI files of these excerpts (each containing at least one harmonically irregular chord), 25 further MIDI files were generated solely by modifying the tones of the irregular chord in a way that this chord became the harmonically most regular, and thus the most expected, chord (always the tonic chord, see example in Figure 1). This procedure was also performed using Cubase SX. Similarly, 25 further MIDI files were generated by rendering the harmonically irregular chord to a very irregular chord (always a Neapolitan sixth chord, see also Figure 1). Thus, there were three versions of each of the 25 excerpts: (1) the original version with the unexpected chord (as arranged by the composer), (2) the version in which this chord was expected, and (3) the version in which this chord was very unexpected, resulting in a total of 75 excerpts. Note that all versions of one excerpt were played with identical emotional expression, and that the only difference between these three versions of each excerpt was the different chord function (expected, unexpected, very unexpected) of the critical chord.

From each of these 75 MIDI files, another MIDI file without emotional expression was created by eliminating all agogics (i.e., variations in tempo), and by adjusting the key-stroke velocity of all notes to the same value, thus eliminating all dynamics (velocity was set to the mean velocity of the corresponding expressive version). Thus, there were 150 different MIDI files in total: 25 excerpts×3 different chord functions (expected, unexpected, very unexpected) ×2 different emotional expressions (expressive, non-expressive).

Audio files of all MIDI files were generated as wav-files using Cubase SX and The Grand (Steinberg). Moreover, in addition to these 150 experimental stimuli, three additional pieces were taken and edited in the same way as described above (resulting in eighteen different MIDI files), with the exception that one chord of the excerpt (occurring in the beginning, the middle or the end of the piece) was played by another instrument than piano (such as marimba, harpsichord, or violin). These timbre-deviants were used for the task of participants during the EEG and SCR recordings (see next section for details).

To evaluate the impact of the stimulus material on individually perceived emotions, a behavioural experiment was conducted with an independent group of subjects. Twenty-five non-musicians (age range 19–30 years, mean age 24.3 years, 12 females) were presented with the 150 experimental stimuli (no stimuli with timbre-deviants were presented, duration of the behavioural testing session was about 45 min). After each excerpt, participants rated how (un)pleasant, aroused, and surprised they felt during the last musical excerpt. Emotional valence (pleasantness) and arousal were assessed using 9-point scales with Self-Assessment Manikins [29], with 1 corresponding to very unpleasant (or very relaxing, respectively) and 9 corresponding to very pleasant (or very arousing, respectively). Surprise was assessed using a 9-point Likert scale, with 1 corresponding to not being surprised at all, and 9 to being very surprised.

**Figure 2:**
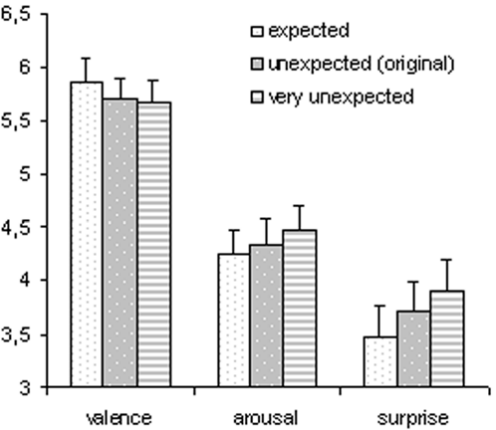
Average ratings of valence, arousal, and surprise, pooled for expressive and non-expressive excerpts (error bars indicate SEM, 1 corresponded to most unpleasant, least arousing, and least surprising, and 9 to most pleasant, most arousing, and most surprising). Ratings differed between the three chord types with regards to valence, arousal, and surprise (see text for details).

Excerpts with expected chords only were rated as most pleasant, least arousing, and least surprising, whereas excerpts with a very unexpected chord were rated as least pleasant, most arousing, and most surprising (ratings for excerpts with an unexpected [original] chord lay between ratings for expected and very unexpected chords with regards to valence, arousal, and surprise, see Table 1 and Figure 2). An ANOVA on the valence ratings with factors chord (expected, unexpected, very unexpected) and expression (expressive, non-expressive) indicated an effect of chord (*F*(2,48) = 12.85, *p*<.0001), an effect of expression (*F*(1,24) = 22.58, *p*<.0001), and no two-way interaction (*p* = .57). Likewise, the analogous ANOVA for the arousal ratings indicated an effect of chord (*F*(2,48) = 7.36, *p*<.005), an effect of expression (*F*(1,24) = 62.31, *p*<.0001), and no two-way interaction (*p* = .48). Finally, the analogous ANOVA for the surprise ratings again indicated an effect of chord (*F*(2,48) = 13.61, *p*<.0001), an effect of expression (*F*(1,24) = 83.02, *p*<.0001), and no two-way interaction (*p* = .23; *p* –values were Greenhouse-Geisser corrected in all three ANOVAs). Paired *t*-tests conducted separately for valence, arousal, and surprise ratings indicated that all six experimental conditions (see Table 1B) differed significantly from each other (*p*≤.05 in all tests), except valence and arousal ratings for unexpected and very unexpected in the expressive condition, and surprise ratings for unexpected and expected in the non-expressive condition.

**Table 1:** Summary of valence-, arousal-, and surprise-ratings (1 corresponded to most unpleasant, least arousing, and least surprising, and 9 to most pleasant, most arousing, and most surprising).

	Valence	Arousal	Surprise
**A**			
Expected	6.08±.21	4.08±.21	3.27±.28
Unexpected (original)	5.86±.21	4.26±.21	3.48±.28
Very unexpected	5.70±.20	4.34±.23	3.71±.28
Expressive	5.67±.21	4.48±.22	3.91±.28
Non-expressive	6.08±.21	3.98±.21	3.06±.28
**B**			
Expressive: expected	5.86±.21	4.34±.23	3.64±.28
Expressive: unexpected	5.68±.20	4.55±.22	3.93±.28
Expressive: very unexpected	5.47±.20	4.57±.24	4.15±.28
Non-expressive: expected	6.29±.22	3.84±.21	2.89±.28
Non-expressive: unexpected	6.04±.23	3.98±.21	3.03±.29
Non-expressive: very unexpected	5.92±.21	4.12±.23	3.27±.28

**A** shows ratings (mean and SEM) averaged across all excerpts with expected chords only, with an unexpected (original) chord, and a very unexpected chord, as well as ratings averaged across all expressive and all non-expressive excerpts. **B** shows ratings (mean and SEM) separately for each of the six experimental conditions.

### Procedure

Participants were informed about the chords played with a deviant instrument, asked to detect such chords, and to indicate their detection by pressing a response button. As examples, two sequences with a deviant instrument were presented before the start of the experiment. The deviant instruments were only employed to control whether participants attended the musical stimulus (this method has already been used in previous studies; e.g., [5], [13], [20], [21], [23]). Participants were not informed about the experimental conditions of interest, i.e. neither were they informed about the different chord functions, nor about the manipulations of emotional expression. During the experimental session, participants were instructed to look at a fixation cross. Each excerpt was followed by a silence interval of 1 s. Each stimulus was presented twice during the experiment to increase the signal-to-noise ratio, the ordering of stimuli was pseudo-randomized. The duration of an experimental session was approximately 70 min.

### Data Recording and Analysis

The EEG was recorded using Ag/AgCl-electrodes from 32 locations of the extended 10–20-system (FP1, FP2, AFz, AF3, AF4, AF7, AF8, Fz, F3, F4, F7, F8, FC3, FC4, FT7, FT8, Cz, C3, C4, T7, T8, CP5, CP6, Pz, P3, P4, P7, P8, O1, O2, nose-tip, and right mastoid), using an electrode placed on the left mastoid as reference. Sampling rate was 500 Hz. After the measurement, EEG-data were re-referenced to the algebraic mean of the left and right mastoid electrodes (to obtain a symmetric reference), and filtered using a 0.25–25-Hz band-pass filter (1001 points, finite impulse response) to reduce artifacts. Horizontal and vertical electrooculograms (EOGs) were recorded bipolarly.

For measurement of the skin conductance response (SCR) two electrodes were placed on the medial phalanx of the index and middle finger of the left hand. For the calculation of the inter-heartbeat-interval (IBI), an electrocardiogram (ECG) was measured by placing two electrodes at the inner sides of the wrists of the left and the right arm.

For rejection of artifacts in the EEG data, each sampling point was centred in a gliding window and rejected if the standard deviation within the window exceeded a threshold value: Artifacts caused by drifts or body movements were eliminated by rejecting sampling points whenever the standard deviation of a 200-ms or 800-ms gliding window exceeded 25 µV at any EEG electrode. Eye artifacts were rejected whenever the standard deviation of a 200-ms gliding window exceeded 25 µV at the vertical or the horizontal EOG (rejections were controlled by the authors). ERPs were calculated using a 200-ms prestimulus baseline.

The electrodermal activity (EDA) data were visually inspected and checked for artifacts caused by movement or failures of the recording device. Data were rejected whenever there was an unusually steep onset of the EDA.

### Data-analysis

For statistical analysis, mean amplitude values were computed for four regions of interest (ROIs): left anterior (F7, F3, FT7, FC3), right anterior (F8, F4, FT8, FC4), left posterior (C3, CP5, P7, P3) and right posterior (C4, CP6, P4, P8).

To test whether ERPs to expected (regular) and unexpected (irregular) chords differ from each other, and whether such differences are lateralized or differ between anterior and posterior scalp regions, amplitude values of ERPs were analyzed statistically by repeated measures ANOVAs. ANOVAs were conducted with factors chord (expected, unexpected, very unexpected), hemisphere (left, right ROIs), and anterior–posterior distribution (anterior, posterior ROIs). Main effects of chord, as well as interactions involving factor chord were adjusted using the Greenhouse-Geisser correction. All statistical analyses of ERPs were computed on the data referenced to the algebraic mean of M1 and M2. The time window for statistical analysis of the ERAN was 140–220 ms, for the N5 500–580 ms. Because the ERAN is defined as the difference between regular (expected) and irregular (unexpected) chords (e.g. [5], [20], [21], [13], [30]), amplitude values and latencies of the ERAN were calculated from the difference ERPs (expected subtracted from unexpected, and expected subtracted from very unexpected chords, respectively). Amplitudes of ERAN and N5 effects (expected subtracted from unexpected, and expected subtracted from very unexpected chords) were tested one-sided according to our hypotheses. To facilitate legibility of ERPs, ERPs were low-pass filtered after statistical evaluation (10 Hz, 41 points, finite impulse response).

## Results

### Behavioural data (timbre detection task)

Participants detected 96.2 percent of the timbre deviants in the expressive, and 95.9 percent in the non-expressive condition (*p* = .98, paired *t*-test), indicating that participants attended to the timbre of the musical stimulus, and that they did not have difficulties in reliably detecting the timbre deviants (neither in the expressive, nor in the non-expressive condition).

### Electrodermal activity

**Figure 3:**
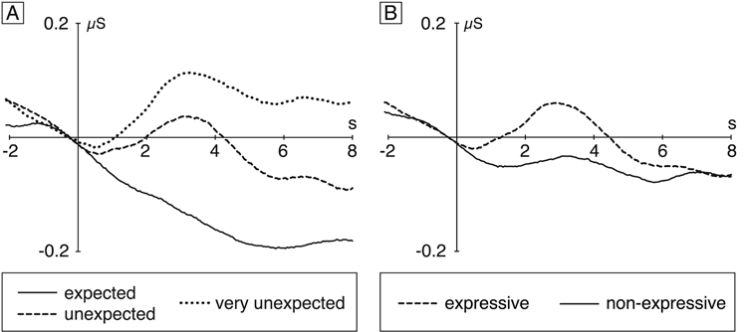
Skin conductance responses (SCRs). A: Grand-average of SCRs elicited by expected, unexpected (original), and very unexpected chords (averaged across expressive and non-expressive conditions). Compared to expected chords, unexpected and very unexpected chords elicited clear SCRs. Notably, the SCR elicited by very unexpected chords was larger than the SCR to unexpected (original) chords, showing that the magnitude of SCRs is related to the degree of harmonic expectancy violation. B: Grand-average of SCRs elicited by expressive and non-expressive chords (averaged across expected, unexpected, and very unexpected conditions). Compared to non-expressive chords, chords played with musical expression elicited a clear SCR.

Figure 3A shows the skin conductance responses (SCRs) to expected, unexpected (original), and very unexpected chords, averaged across all subjects (and across both expressive and non-expressive conditions). Compared to expected chords, both unexpected and very unexpected chords elicited a tonic SCR with an onset of around 500 ms, the SCR being largest for very unexpected chords. Figure 3B shows the SCRs separately for all chords played with and without expression. The expressive chords elicited a more phasic SCR which was stronger than the SCR to non-expressive chords, the SCR amplitude being maximal at around 2.5 seconds.

A global ANOVA with factors chord (expected, unexpected, very unexpected) and expression (expressive, non-expressive) for a time window ranging from 1.5 to 3.5 sec indicated an effect of expression (*F* (1,19) = 5.17, *p*<0.05, reflecting that expressive chords elicited a stronger SCR than non-expressive chords), a marginal effect of chord (*F* (2,38) = 2.53, *p* = 0.09, reflecting that SCRs to very unexpected, unexpected, and expected chords differed from each other), but no two-way interaction (*p* = .79). The effect of chord was significant (*F* (1,19) = 4.66, *p*<.05) when an analogous ANOVA was conducted for a longer time window (1.5 to 8 s, justified because the effect of chord was more tonic than the effect of expression, see Figure 3A vs. 3B). Two-tailed paired *t*-tests showed that this effect of chord was due to a significant difference in SCRs between very unexpected and expected chords (*p*<0.05), and between very unexpected and unexpected chords (*p*<.05). Although clearly visible in the waveforms, the difference in SCRs between unexpected and expected chords was statistically not significant (*p* = .22).

Although the ANOVAs did not indicate an interaction between factors chord and expression, we also inspected the single subject data sets for differences in SCR effects between the expressive and the non-expressive conditions (to exclude that the large variance typical for SCR data rendered the results of the ANOVA spurious). In 16 (out of 20) participants the difference in SCRs between very unexpected and expected chords was larger for the expressive than for the non-expressive chords, but only 9 participants showed this effect of expression for the difference between unexpected (original) and expected chords. A Chi-Square test on the SCR effects of very unexpected chords (SCRs to expected chords subtracted from SCRs to very unexpected chords in the time window from 1.5 to 3.5 s), indicated that significantly more subjects displayed a larger SCR when chords were played with expression than when they were played without expression (*X*(1) = 7.2, *p*<0.01).

### Heart rate

There were no significant differences in the inter-heartbeat interval (IBI) following the presentation of the three types of chords (expected, unexpected, very unexpected) in any of the three time windows (0–2 sec: *p* >.7; 2–4 sec: *p* >.3; 4–6 sec: *p* >.3), and the IBIs were identical when calculated for entire expressive and non-expressive excerpts (0.92 sec in each condition).

### Electroencephalogram

#### ERAN

**Figure 4:**
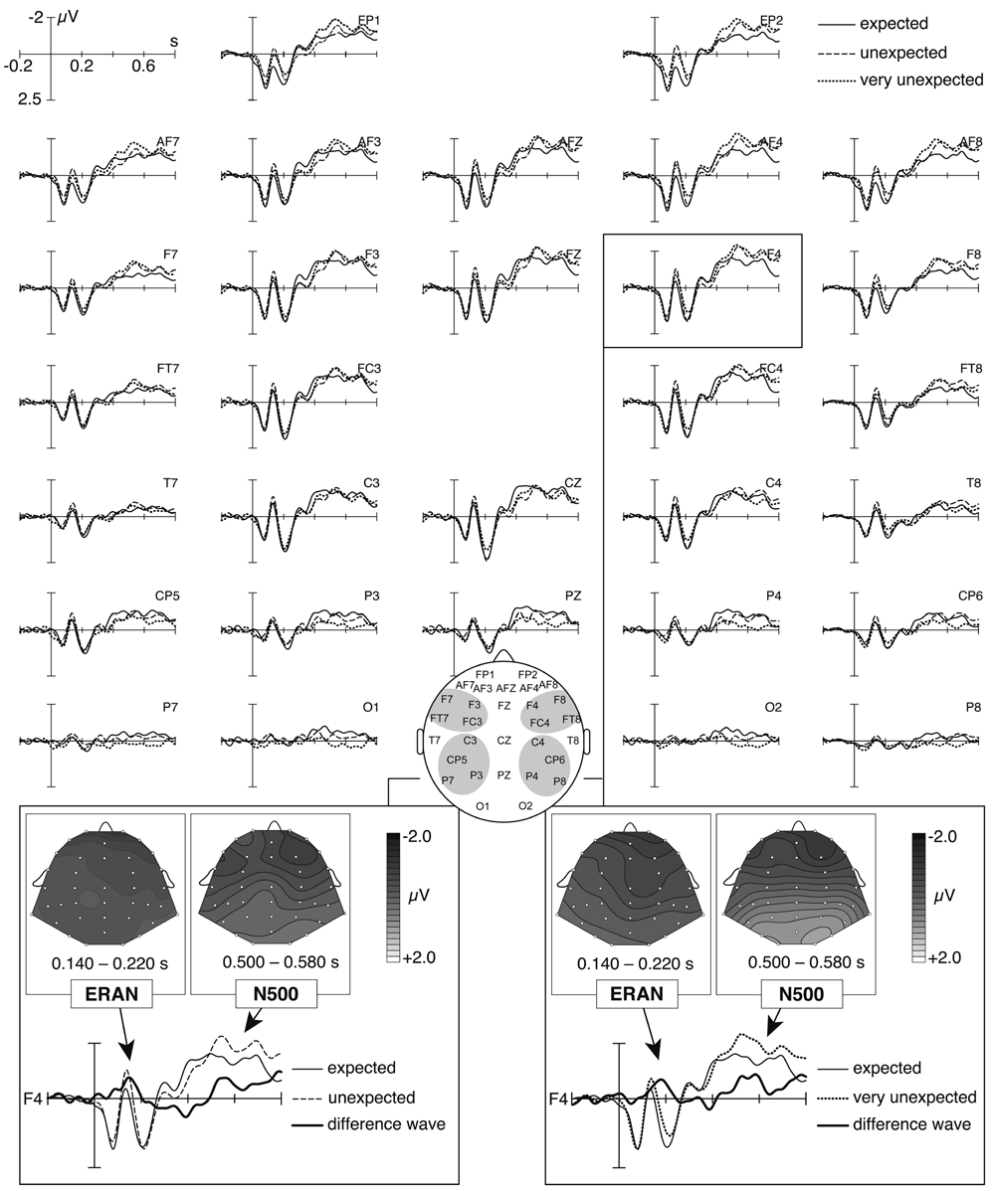
Grand-average of brain electric responses to expected, unexpected (original), and very unexpected chords (averaged across expressive and non-expressive conditions). Compared to expected chords, both unexpected and very unexpected chords elicited an ERAN and an N5. The insets in the two bottom panels show isopotential maps of the ERAN and the N5 effect (expected subtracted from [very] unexpected chords).

Compared to the expected chords, both unexpected (original) and very unexpected chords elicited an ERAN (Figure 4). The peak latency of the ERAN elicited by unexpected chords (expected subtracted from unexpected chords) was 158 ms, and 180 ms for the ERAN elicited by very unexpected chords (expected subtracted from very unexpected chords). As expected, the ERAN elicited by very unexpected chords was nominally larger (−0.84 µV) than the ERAN elicited by unexpected chords (−0.74 µV), but this difference was statistically not significant (amplitude values were calculated for frontal ROIs in the time window from 140 to 220 ms as difference potentials, expected subtracted from [very] unexpected chords). Moreover, the ERAN was identical for the expressive and the non-expressive condition (−0.79 µV in each condition).

A global ANOVA with factors chord (expected, unexpected, very unexpected), expression (expressive, non-expressive), anterior- posterior distribution, and hemisphere for a time window from 140 to 220 ms indicated an interaction between factors chord, anterior-posterior and hemisphere (*F* (2,38) = 3.6, *p*<.05, reflecting that the ERAN amplitude was largest at right anterior leads). A follow-up ANOVA with factors chord and expression for the right anterior ROI indicated an effect of chord (*F* (2,38) = 4.01, *p*<.05; reflecting that ERPs elicited by expected, unexpected, and very unexpected chords differed from each other), but no effect of expression (*p* = .26), and no two-way interaction (*p* = .73). Paired *t*-tests for the anterior ROI indicated that both unexpected (compared to expected) and very unexpected (compared to expected) chords elicited an ERAN (both *p*<.05). A paired *t*-test for the anterior ROI comparing directly the ERP amplitudes of unexpected and very unexpected chords did not indicate a difference (*p* = 0.36).

#### N5

**Figure 5:**
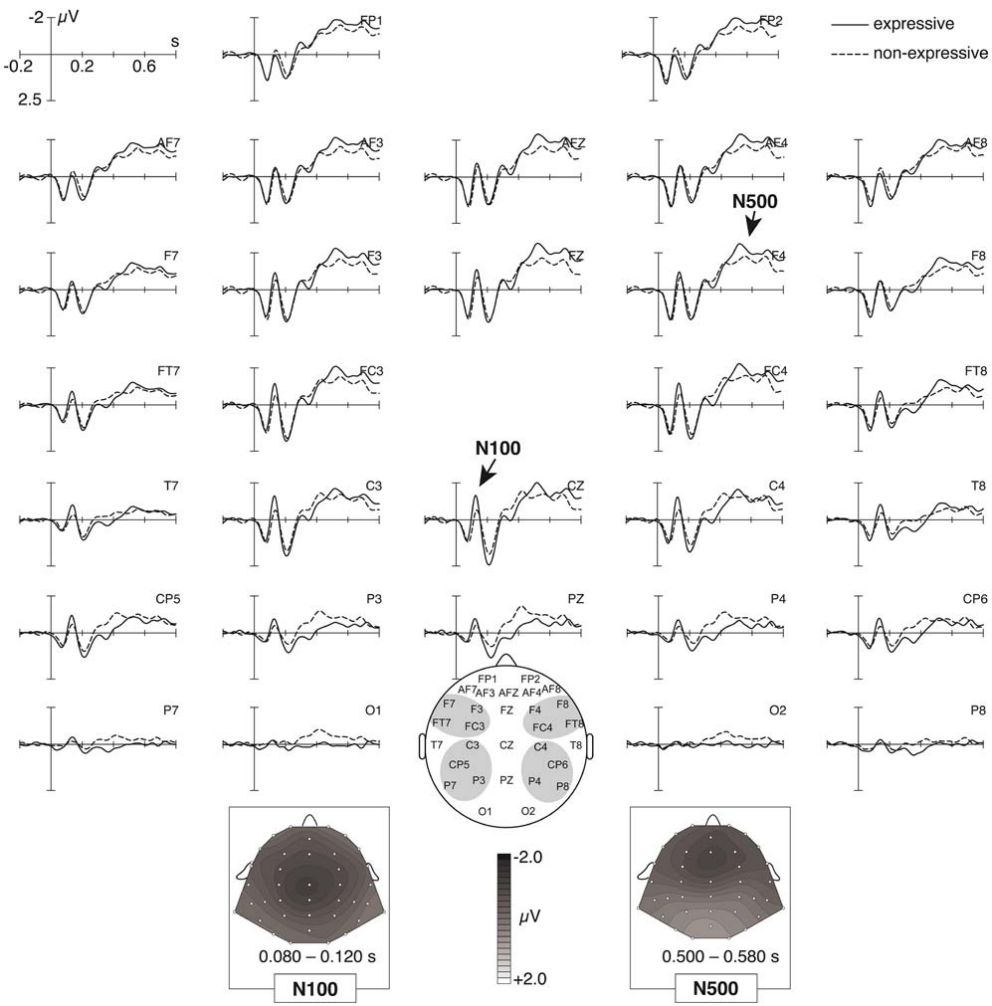
Grand-average of brain electric responses to expressive and non-expressive chords (averaged across expected, unexpected, and very unexpected conditions). Expressive chords elicited a negative effect in the N100-range (being maximal at central electrodes), and an N5 that was larger than the N5 elicited by non-expressive chords. The bottom insets show isopotential maps of the N1 and N5 effect (non-expressive subtracted from expressive chords).

In the ERPs of both unexpected and very unexpected chords, the ERAN was followed by a late negativity (the N5, Figure 4). Compared to expected chords, the N5 was nominally larger for very unexpected than for unexpected (original) chords, but this difference was statistically not significant. The amplitudes of the N5 effects (unexpected or very unexpected chords compared to expected chords) did not clearly differ between the expressive and the non-expressive condition. Interestingly, when comparing all expressive chords to all non-expressive chords, the N5 was larger for expressive chords (Figure 5).

A global ANOVA with factors chord (expected, unexpected, very unexpected), expression, anterior-posterior distribution, and hemisphere for the time window from 500 to 580 ms indicated an interaction between chord and anterior-posterior (*F* (1,19) = 13.77, *p*<0.0001, reflecting that the N5 was larger over anterior than over posterior regions). The analogous ANOVA for anterior ROIs indicated significant effects of chord (*F* (2,38) = 2.96, *p*<0.05, tested one-sided according to our hypothesis) and of expression (*F* (1,19) = 7.27, *p*<0.02), but no interaction between factors chord and expression (*p* = .17). Paired *t*-tests for the anterior ROIs indicated that both unexpected (compared to expected) and very unexpected (compared to expected) chords elicited significant effects (both *p*<.05). A paired *t*-test for the anterior ROI comparing directly the ERP amplitudes of unexpected and very unexpected chords did not indicate a difference (*p* = 0.76).

#### P3a

Following the ERAN, the ERPs of both unexpected and very unexpected chords compared to expected chords were more positive around 300 ms, particularly over left anterior leads (see Figure 4). To test whether this difference reflects the elicitation of a P3a (see *Discussion* for functional significance of the P3a), an ANOVA was conducted with factors chord (expected, unexpected, very unexpected), expression, and hemisphere for anterior ROIs and a time window from 250–350 ms. This ANOVA did not indicate an effect of chord (*p* = .72), nor any no two- or three-way interaction, indicating that unexpected or very unexpected chords did not elicit significant P3a effects.

#### Expressive vs. non-expressive chords

Figure 5 shows ERPs elicited by all chords in the expressive and the non-expressive condition. In addition to the larger N5 elicited by expressive compared to the non-expressive chords (see above), the expressive chords also elicited an increased negativity over central electrodes in the time window from 80 to 120 ms (this effect is presumably an increased N100 due to the fact that the critical chords were usually played more loudly in the expressive condition, see also *Discussion*).

An ANOVA for the time-window from 80 to 120 ms with factors expression, anterior-posterior distribution, and hemisphere did not indicate an effect of expression (*p* = .16). However, an ANOVA computed for central electrodes only (T7, T8, C3, C4) with factor expression (time window 80 to 120 ms) indicated a significant effect (*F* (1,19) = 4.70, *p*<.05).

## Discussion

### Electrodermal activity and heart rate

Compared to expected chords, very unexpected chords elicited a significant skin conductance response (SCR). A smaller SCR effect was clearly observable for unexpected (original) chords, although this effect was statistically not significant. These SCR data replicate findings from a previous study [18], suggesting that unexpected harmonies elicit an emotional response, and that the strength of this response increases with increasing unexpectedness of a harmony. The notion that the SCRs to (very) unexpected chords reflect effects of emotional processing is also supported by the emotion ratings: These ratings showed that excerpts with expected chords differed from those with an unexpected, and even more so from those with a very unexpected chord in terms of emotional valence, arousal, and surprise. Our findings hence lend further support to the theory of Meyer [25] that violations of harmonic expectancy elicit emotional responses in listeners (such as tension-relaxation or surprise; see also [26]).

It is unlikely that the SCRs elicited by the (very) unexpected chords were simply due to attentional mechanisms or orienting reflexes which could have been triggered by these chords, because the ERP analysis showed that neither unexpected nor very unexpected chords elicited a significant P3a. Attentional mechanisms and orienting reflexes are usually reflected in P3a potentials (e.g, [31]), even when subjects do not have to attend to a stimulus, or a stimulus dimension. If the significant SCR to very unexpected chords (as well as the SCR to unexpected chords) could be explained by attention-capturing mechanisms (or orienting reflexes) triggered by such chords, then a significant P3a should have been observable in the ERPs, which was not the case.

The SCRs to all chords played with expression were larger compared to the SCRs to all non-expressive chords. Note that, originally, the expressive chords were all slightly unexpected chords as arranged by the composer (and these original versions with unexpected chords were the ones played by the pianists). To increase the emotional response to such unexpected harmonies, performers use means of emotional expression in music, such as playing the notes with increased or decreased key-stroke velocity (e.g., an accent, or an unexpectedly soft timbre). Thus, when rendered expected, or very unexpected, the critical chords in the expressive conditions all differed in their key-stroke velocity compared to the preceding chords (most of them being played with an accent, i.e. with increased loudness), whereas all notes in the non-expressive condition were played with the same key-stroke velocity as all other chords. It is probable that this increased loudness of the expressive chords led to the increased SCR (as well as to the increased N100 amplitude to expressive chords). With regards to the SCR, an alternative explanation is that all chords in the expressive condition elicited stronger electrodermal activity as a function of being more emotionally expressive and eliciting the appropriate emotion-related response in the listener (consistent with the behavioural data showing that expressive excerpts were perceived as more arousing than non-expressive excerpts); this issue remains to be specified.

It is not possible that the SCRs to expressive chords were larger simply because of increased resources required to discern the timbre deviants, or because of increased expectancies for the timbre deviants during the expressive condition (which could have led to increased tonic levels of electrodermal activity): First, general differences in processing demands would have been reflected in the skin conductance level across entire excerpts (and thus not be visible in the SCRs to chords that were calculated using a baseline preceding the chords). Second, the behavioural data of the timbre detection task indicate that this task was comparably easy in both expressive and non-expressive conditions. Finally, the heart rate calculated for entire excerpts (reflecting the vagal tone during listening to the excerpts) was identical for expressive and non-expressive excerpts, rendering it unlikely that the task of detecting the timbre deviants was more engaging, or more attention-demanding, in the expressive condition.

Interestingly, the SCRs elicited by very unexpected chords (compared to expected chords) tended to be more pronounced when these chords were played with expression compared to when played without expression. That is, it appears that the emotional response elicited by a (very) unexpected harmony can be enhanced when perceived in a musical context played with emotional expression (using variations in loudness and tempo). Note that purely physical differences between expressive and non-expressive conditions cannot account for this effect, because we compared the difference in SCRs between expected and (very) unexpected harmonies, separately for the expressive, and for the non-expressive music.

No significant changes in the inter-heartbeat interval (IBI) for the three different types of chords were observed (as in a recent study by Steinbeis et al. [18]), and no differences in IBIs were measured between expressive and non-expressive chords. We surmise that the lack of IBI changes is due to the short duration of the events of interest (i.e., of the chords), and perhaps due to the relatively small differences in emotional valence between chords (although these differences were statistically significant in the behavioural data). Thus, SCRs (which differed between expected, unexpected, and very unexpected chords) appear to be more suitable to investigate sympathetic effects of emotional responses to harmonic irregularities in music.

### Electroencephalogram

#### ERAN

Compared to the expected chords, unexpected (original) chords elicited an ERAN. This is the first evidence showing that unexpected chords, as arranged by a composer, and as played by pianists, elicit an ERAN. Previous studies have either used rather artificial musical stimuli (see Introduction) to have the maximum control over the acoustic properties of the stimuli, or used original excerpts which were played by a computer [18], or used excerpts played by a musician, but with unexpected musical events that were arranged by the experimenters (and not by the composer [15], [16]). The present data, thus, show brain responses to authentic musical stimuli, i.e., to music as it was actually composed by a composer, and played by a pianist. The ERAN elicited by very unexpected chords was nominally larger than the ERAN elicited by unexpected chords (as hypothesized, although this difference was statistically not significant). This tentatively replicates results of previous studies [5], [18] showing that the amplitude of the ERAN increases with increasing harmonic irregularity, and thus unexpectedness, of a chord.

Note that it is unlikely that the ERAN is simply an attention effect on the N100 component, because the ERAN latency was around 160–180 ms, which is well beyond the N100 latency. Also note that, due to its slightly larger magnitude, the ERAN elicited by the very unexpected chords extended into the P2-range, which is in agreement with a number of previous studies [5], [20], [21], [13], [30].

Importantly, the ERAN did not differ between the expressive and the non-expressive condition. That is, the ERAN did not differ when elicited in a musical context played with emotional expression compared to when played without emotional expression. This indicates that music-syntactic processing (as indicated by the ERAN) does not interact with the emotional expression of a musical excerpt. That is, the generation of the ERAN appears to be independent of the increased emotional response elicited by an unexpected harmony played with emotional expression (as indicated by the SCRs and the behavioural data). This suggests that the neural mechanisms of music-syntactic processing operate independently of the emotional factors communicated by musical performance. Note that the outcome of music-syntactic processing (which leads to the perception of unexpectedness of a harmonically irregular chord) has clear effects on emotional processes (as shown by the SCRs to [very] unexpected chords, which were stronger than those to expected chords, see also above). The finding that the ERAN did not differ between the expressive and non-expressive condition justifies the use of non-expressive musical stimuli in other studies on music-syntactic processing in order to have the maximum acoustic control over the stimulus.

#### N5

In both the expected and the very unexpected condition, the ERAN was followed by a late negativity, the N5 [5], [20]–[23]. The N5 is taken to reflect processing of harmonic integration which is at least partly related to the processing of musical meaning [24], [5]. For example, Steinbeis and Koelsch [32] showed an interaction between the N5 and the N400 (elicited by semantic incongruities in language), suggesting that the integration of expected and unexpected events into a larger, meaningful musical context consumes partly resources that are also engaged in the processing of linguistic semantics.

The amplitude of the N5 effect (expected subtracted from [very] unexpected chords) did not clearly differ between the expressive and the non-expressive condition. However, the N5 potentials clearly differed between all expressive and all non-expressive chords (Figure 5). With regards to this, it is important to note that the N5 is not only elicited by harmonically irregular (unexpected) chords, but also by harmonically regular (expected) chords [5, 22; see also Figure 4]: Although irregular chords usually require a higher amount of harmonic integration (leading to a larger N5), regular chords also require integration into the harmonic context. Thus, both irregular (unexpected) and regular (expected) chords may elicit N5 potentials (the N5 elicited by unexpected chords usually being larger than the N5 evoked by expected ones, see Figure 4). The finding that the N5 (averaged across expected, unexpected, and very unexpected chords) was larger for chords played with emotional expression compared to chords played without such expression indicates that the N5 can be modulated by emotional expression. The reason for this modulation is presumably that chords played with expression contain more meaning information (because they contain information about the emotions and the intentions of the performer, [27]), resulting in larger N5 potentials for expressive than for non-expressive chords. Notably, no effect of expression was found in the ERAN time window. Thus, whereas the ERAN is not influenced by emotional expression of a musical excerpt, the neural mechanisms underlying the generation of the N5 can be influenced by emotional expression.

### Conclusions

Our data show a number of physiological responses elicited by music played as naturally as one would encounter in real-life listening situations. Unexpected harmonies (as actually arranged by composers) elicited both ERAN and N5 potentials. A number of previous studies used musical sequences that were played without expression under computerized control, raising the question whether ERAN and N5 are simply experimental artefacts. The data from the expressive condition show that the neural mechanisms underlying the generation of both ERAN and N5 are also involved in the processing of real naturalistic music. The SCRs elicited by very unexpected chords (compared to expected chords), tended to be influenced by the emotional expression of the musical performances, suggesting that emotional effects of the processing of unexpected chords are slightly stronger when elicited by expressive music. By contrast, the ERAN was not modulated by emotional expression. This suggests that the neural mechanisms of music-syntactic processing operate independently of the emotional factors communicated by musical performance, and that the ERAN is, thus, a cognitive process which is independent of the emotional qualities of a stimulus. This justifies the use of stimuli without emotional expression to investigate the cognitive processing of musical structure. Notably, the outcome of music-syntactic processing (as reflected in the ERAN) leads to the perception of unexpectedness of a harmonically irregular chord, and has clear effects on emotional processes as reflected by the SCRs to unexpected chords. The N5 elicited by chords in general (regardless of their chord function) was modulated by emotional expression, presumably because chords played with expression contain additional meaning information about the emotions and the intentions of the performer. Thus, our data also indicate that musical expression affects the neural mechanisms underlying harmonic integration and processing of musical meaning.

Thus, the present results suggest that (a) the neural mechanisms underlying the generation of both ERAN and N5 are involved in the processing of real naturalistic music, (b) music-syntactic processing as reflected in the ERAN is a cognitive process which is independent of the emotional qualities of a stimulus, (c) emotional effects of the processing of unexpected chords are slightly stronger when elicited by expressive music, and (d) that musical expression affects the neural mechanisms underlying harmonic integration and processing of musical meaning.
